# Association between Obesity, Serum Lipids, and Colorectal Polyps in Old Chinese People

**DOI:** 10.1155/2013/931084

**Published:** 2013-10-01

**Authors:** Wen Yang, Yan Chang, Haiyan Huang, Yuzhu Wang, Xiaohong Yu

**Affiliations:** Geriatric Digestive System Department, Navy General Hospital, No. 6 Fuchenglu Road, Beijing 100048, China

## Abstract

*Background*. Colorectal cancer mostly arises from the polyps of colon. The aim of our study was to examine the association of body mass index (BMI) and serum lipids with the colorectal polyps in old Chinese people. *Methods*. The risk of developing colorectal polyps was studied in 244 subjects (212 men and 32 women, 74.63 ± 11.63 years old) who underwent colonoscopy for the first time from January 2008 to July 2012 at the Navy General Hospital, Beijing, China. According to the results of colonoscopy, all the subjects were divided into 112 normal control, 38 right colorectal polyps, 53 left colorectal polyps, and 41 both right and left colorectal polyps groups. The total plasma cholesterol, plasma triglyceride, plasma creatinine concentration, blood urinary nitrogen, and fasting glucose were determined using a multichannel analyzer. *Results*. There were significant differences among normal control, right colorectal polyps, left colorectal polyps, and both right and left polyps groups, which were the BMI, total cholesterol, triglycerides, creatinine, and urinary nitrogen. In binary logistic regression analysis, there were two risk factors associated with the occurrence of colorectal polyps, which included BMI and systolic blood pressure. *Conclusions*. Colorectal polyps were significantly associated with increased BMI, total cholesterol, and triglycerides levels.

## 1. Introduction

According to a report of the World Health Organization, cancer was the leading cause of death in 2007, accounting for 7.9 million deaths, or 13% of the total amount. The same report stated that colorectal cancer was the fourth most common fatal cancer, after lung, stomach, and liver cancers [1]. Epidemiological evidence has shown that obesity is associated with an increased risk of mortality from cancers [2, 3]. In some studies, serum triglyceride [4–7] and cholesterol [8–10] levels are positively related to an increased risk of colorectal adenoma, while several investigators report an insignificant or even inverse relationship between serum lipids and colorectal adenoma [11–13]. The prevalence of obesity and dyslipidemia is rising dramatically in both developed and developing countries. Therefore, we examined the association between body mass index (BMI), serum lipids, carcinoembryonic antigen (CEA), carbohydrate antigen 19-9 (CA19-9), and colorectal polyps in relatively immobile resident of old Chinese people, hoping to provide useful information for preventing colorectal cancer.

## 2. Methods

### 2.1. Study Population

The subjects comprised 244 individuals who underwent a routine screening colonoscopy at the navy general hospital, Beijing, china, between January 2008 and July 2012. There were 212 men (average age, 75.27 ± 11.68 years) and 32 women (average age, 70.38 ± 10.53 years). For the analysis of the occurrence of colorectal polyps, we included patients with the following conditions: complete, available medical records; normal blood pressure (diastolic blood pressure of less than 90 mmHg and systolic blood pressure of less than 150 mmHg) or well-controlled hypertension (diastolic blood pressure of less than 90 mmHg and systolic blood pressure of less than 150 mmHg). The hypertensive patients were taking antihypertensive medication, and all the observed patients had taken acetylsalicylic acid (100 mg/day). Exclusion criteria were as follows: using medication for hyperlipidemia and other* NSAIDs *(except acetylsalicylic acid); past history of cancer, coronary artery disease, diabetes mellitus, inflammatory bowel disease, familial polyposis or thyroid disease; major gastrointestinal surgery, including partial or total gastrectomy or colectomy; colon cancer diagnosed during sigmoidoscopic examination; vegetarian patient; dieting; liver cirrhosis or SGPT levels three times higher than the normal limit; nephrotic syndrome or serum creatinine levels higher than 115 umol/L; The CEA levels higher than 10 ng/mL; incomplete examination and missing data.

Hypertension was defined as a systolic blood pressure (SBP) ≧ 140 mmHg or diastolic blood pressure (DBP) ≧ 90 mmHg according to the Seventh Report of the Joint National Committee [14] or when the subjects had a history of hypertension or were receiving antihypertensive treatment.

The diagnostic criteria for coronary artery disease included the previous onset of myocardial infarction or more than 75% narrowing of the coronary artery, as determined by radiography.

The protocol of the present study was reviewed and approved by the Ethics and Research Committee of the Navy General Hospital (Beijing, China). The study population gave informed consent before blood sampling.

### 2.2. Diagnosis of Colorectal Polyps

Endoscopists were physicians who had been trained for 6 months or longer at our hospital and had carried out 500 or more colonoscopies. Each endoscopist carried out six or more colonoscopies per day. Insertion up to the cecum was successful in 96.8% of examinations. Digital examination of the rectum was routine prior to the insertion of the endoscope. In preparation for colonoscopy, subjects took a laxative (polyethylene glycol electrolyte powder, BEAUFOUR IPSEN Industrie, Dreux, France) on the night before the examination and on the day of colonoscopy at 08:30 h (colonic lavage using approximately 2000 mL of solution was carried out). Small-caliber electronic colonoscopies (CF-200I, 240I, 240AI, and Q260AI; Olympus, Tokyo, Japan) were used for colonoscopy. All visualized lesions were biopsied and histologically assessed by experienced pathologists.

**Figure 1:**
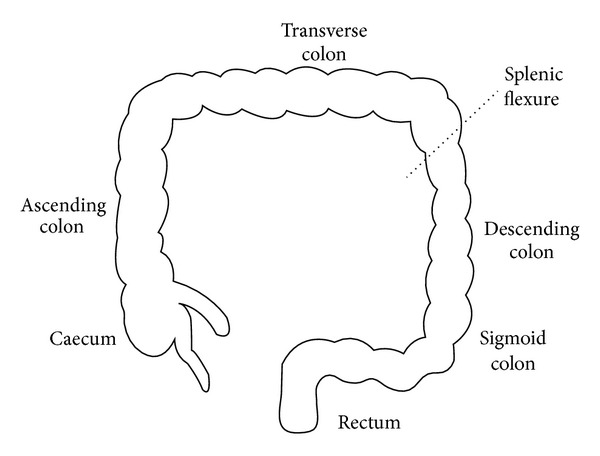


For the purpose of our analysis, colorectal polyps which arise proximal (right) or distal (left) to the splenic flexure were divided into three groups [15], which are right colon group, left colon group, and both right and left colon group, and comparative analysis for the clinical and endoscopic data among the three groups and a multiple comparison were conducted (Figure 1).

### 2.3. Metabolic Risk Factors and Other Factors

The blood samples were drawn from participating patients after an overnight fast of more than 12 h, and also they stopped smoking, drinking alcohol, tea, or coffee. The serum levels of plasma creatinine concentration, blood urinary nitrogen, total plasma cholesterol, plasma triglyceride were measured using a multichannel analyzer (Roche Hitachi 737; Boehringer Mannheim Diagnostics, USA).

The CEA and CA19-9 levels were measured by means of immunometric chemiluminescent assay kit (Beckman Co, USA).

### 2.4. Anthropometric Measurements

Anthropometric measurements were made by well-trained examiners on individuals wearing light clothing and without shoes. Height was measured to the nearest 0.1 cm and weight to the nearest 0.1 kg; body mass index (BMI) was calculated for these patients as weight (in kilograms) divided by the square of height (in meters).

### 2.5. Statistical Analysis

Data were expressed as mean ± SD or counts. Statistical analysis was performed using SPSS version 16.0 (SPSS Inc, USA), and the level of statistical significance was defined as *P* < 0.05. Tests for several independent samples were used to explore the association between different categories of colorectal polyps groups and sex, hypertension, hyperplastic polyp, tubular adenoma, and tubulovillous adenoma. The one-way ANOVA was used for others appropriate factors between different categories of colorectal polyps. Binary logistic regression analysis was used to determine the factors associated with colorectal polyps in the entire population. 

## 3. Results

### 3.1. Baseline Characteristics

All the patients were divided into four groups based on their colorectal polyps: normal, right colorectal polyps, left colorectal polyps, and both right and left colorectal polyps. The 244 patients were evaluated at baseline and found to be eligible for the present analysis. The demographic characteristics of these people are summarized in Table 1.

### 3.2. Associations of BMI, Total Cholesterol, Triglycerides, Fasting Glucose, Creatinine, and Urinary Nitrogen Levels with the Presence of Colorectal Polyps

One-way ANOVA post hoc tests showed that the BMI (*P* < 0.05), total cholesterol (*P* < 0.01), triglycerides (*P* < 0.01), creatinine (*P* < 0.01) and urinary nitrogen (*P* < 0.05) were the significant differences among normal control, right colorectal polyps, left colorectal polyps, and both right and left polyps groups (Table 2). The BMI of both right and left polyps group (24.91 ± 2.64) was significantly different from the normal control groups (23.14 ± 2.03) (*P* < 0.01); the total cholesterol of both right and left polyps group (4.92 ± 1.25) was significantly different from the normal control groups (4.01 ± 0.80) (*P* < 0.01), right colorectal polyps group (4.04 ± 1.02) (*P* < 0.01), and left colorectal polyps group (4.31 ± 0.90) (*P* < 0.01); the total cholesterol of left colorectal polyps group was significantly different from the normal control group (*P* < 0.05). The triglycerides of both right and left polyps group (1.65 ± 1.15) were significantly difference from the normal control groups (1.06 ± 0.49) (*P* < 0.01) and right colorectal polyps group (1.14 ± 0.59) (*P* < 0.05); the triglycerides of left colorectal polyps group (1.65 ± 1.48) were significantly different from the normal control groups (*P* < 0.01), right colorectal polyps group (*P* < 0.05). The fasting glucose of both right and left polyps group (6.07 ± 2.47) was significantly different from the right colorectal polyps group (5.40 ± 0.96) (*P* < 0.05). The creatinine of both right and left polyps group (103.15 ± 26.48) was significantly different from the right colorectal polyps group (118.68 ± 45.27) (*P* < 0.05); the creatinine of left colorectal polyps group (94.88 ± 21.36) was significantly different from the right colorectal polyps group (118.68 ± 45.27) (*P* < 0.01); the creatinine of right colorectal polyps group (118.68 ± 45.27) was significantly different from the normal control group (99.68 ± 29.38) (*P* < 0.01). The urinary nitrogen of left colorectal polyps group (5.69 ± 2.16) was significantly different from the right colorectal polyps group (6.98 ± 2.98); the urinary nitrogen of right colorectal polyps group was significantly different from the normal control group (6.14 ± 1.37) (*P* < 0.05).

The comparison of female, hyperplastic polyp, tubular adenoma, tubulovillous adenoma, and hypertension among the four groups was not a significant difference, which was analysed by tests for several independent samples (*P* > 0.05).

### 3.3. The Relationship of Colorectal Polyps and Other Factors

In the binary logistic regression analysis, in which colorectal polyps were taken as a dependent variable and age, BMI, total cholesterol, triglycerides, fasting glucose, creatinine, urinary nitrogen, systolic blood pressure, and diastolic blood pressure were taken as covariates, we found that there were two risk factors associated with the occurrence of colorectal polyps, which included BMI (OR = 1.641; 95% CI: 1.022–2.635, *P* < 0.05) and systolic blood pressure (OR = 0.85; 95% CI: 0.758–0.953, *P* < 0.01) (Table 2). 

## 4. Discussion

A number of reports have demonstrated that obesity is a risk factor for colorectal polyps (both adenomatous and hyperplastic polyps) and cancer in men [16–20]. Our study showed that the association between BMI and colorectal polyps was significant in the unadjusted analysis, which is in agreement with the previous studies.

The mechanisms underlying the association between BMI and colorectal polyps and its role in predicting colorectal polyps in obesity patients are unclear. There are two possible mechanisms that might explain our findings. First, recently, insulin and insulin-like growth factors (IGF) have been suggested to play a role in colorectal carcinogenesis as the underlying mechanism for the increased incidence in obese subjects [21, 22]. It is known that both molecules have a role in cell proliferation via the receptors present in normal and cancer cells of the large bowel. In particular, visceral fat obesity via this mechanism induces hyperinsulinemia and high IGF-1 concentrations in the blood, which is thought to promote proliferation and division of large bowel cells and induce carcinogenesis [21–23]. Second, the gene-encoding leptin has been linked to the growth and development of cancer [24]. Leptin has been shown to regulate neoangiogenesis by itself and in concert with vascular endothelial growth factor and fibroblast growth factor 2. In addition to its proangiogenic activity, leptin can enhance endothelial cell growth and suppress apoptosis through a Bcl-2-dependent mechanism and can act as a mitogen, transforming, or migration factor for many different cell types [25]. In particular, leptin may be directly involved in colon tumorigenesis, or it may serve as a sensitive and robust marker of an obesity-induced adverse endocrine environment [26, 27]. 

In our study, patients with colorectal polyps were more likely to have high total cholesterol, triglycerides, fasting glucose, and urinary nitrogen levels; total cholesterol and triglycerides were associated with the site and number of colorectal polyps. One study by Tabuchi et al. performed a large scale retrospective study with 4,887 patients to analyze the correlation between the incidence of colorectal adenoma and serum levels of total cholesterol and triglyceride. Multiple logistic regression analysis with adjustment for age and gender revealed that triglyceride was an independent correlation factor in males with tubular adenoma but not with villous adenoma [28]. Most studies show that triglyceride level positively related to an increased risk of colorectal adenoma polyps [28, 29], but the studies of cholesterol levels have different results. Some studies of cholesterol levels are positively related to an increased risk of colorectal adenoma, while some are not related. Our research papers show that cholesterol levels are related to an increased risk of colorectal adenoma. 

The possible mechanisms of relationship between colorectal polyps and serum lipids might explain our findings. First, hypertriglyceridemia is associated with hyperinsulinemia and insulin resistance [30, 31], and hyperinsulinemia and insulin resistance can induce colorectal polyps as above expatiated. Second, serum triglyceride concentration may be positively associated with bile acid synthesis and fecal bile acids. The increase of synthesized and secreted bile acids may provide abundant substrates for the formation of secondary bile acids and promote carcinogenesis in the large bowel [32].

About the relationship of colorectal polyps and fasting glucose levels, one possible explanation is that fasting glucose levels were related to obesity or serum lipids, so that fasting glucose levels were increased as obesity or serum lipids. 

This study had several limitations. There may have been a selection bias in that subjects in this study were recruited from individuals who visited the hospital for regular health examination and underwent colonoscopies; thus, they were more concerned about their health status and were of a higher socioeconomic status than the general population. Approximately 54% of these individuals had colorectal polyps, an incidence higher than that in other studies [33, 34]. In addition, as this study was mostly on men and limited to old people, we should speculate whether the results may be generalized to all population.

The current study provides new evidence linking the obesity and serum lipids to colorectal polyps' etiology and shows a positive association between obesity and colorectal polyps risk as well as between total cholesterol, triglycerides, and colorectal polyps risk in old Chinese people. To consider the role of dietary fat as well as serum lipids in carcinogenesis, more detailed studies are needed to clarify the effects of both systemic and local effect of lipid constituents on gastrointestinal cancer development. Further investigations in this subject should also take into account other metabolic determinants including insulin and gut microbiota.

## Figures and Tables

**Table 1:** Comparisons of demographic and clinical characteristics among the four groups.

Variables	Normal control	Right colorectal polyps	Left colorectal polyps	Both right and left polyps	*P* value
	112	38	53	41	
Age (years)	74.88 ± 11.48	75.32 ± 11.39	71.51 ± 12.08	77.34 ± 11.20	0.101
Female (*n*)	17	3	7	5	0.717
Hyperplastic polyp (*n*)		7	15	3	1.000
Tubular adenoma (*n*)		30	35	33	1.000
Tubulovillous adenoma (*n*)		1	3	3	1.000
BMI (kg/m^2^)	23.14 ± 2.03	23.67 ± 3.21	24.05 ± 5.611	24.91 ± 2.64^a^	0.034
Hypertension (*n*)	32	9	11	9	0.520
SBP (mmHg)	131.23 ± 12.90	130.74 ± 14.23	131.81 ± 11.84	129.78 ± 12.58	0.889
DBP (mmHg)	74.60 ± 9.37	73.24 ± 8.27	74.36 ± 9.11	74.73 ± 9.05	0.867
CEA (ng/mL)	2.30 ± 1.35	2.35 ± 1.08	2.02 ± 1.28	2.20 ± 2.15	0.699
CA19-9 (U/mL)	13.35 ± 15.19	16.14 ± 14.00	17.42 ± 17.36	18.56 ± 30.55	0.397
Total cholesterol (mmol/L)	4.01 ± 0.80	4.04 ± 1.02	4.31 ± 0.90^b^	4.92 ± 1.25^ace^	0.000
Triglycerides (mmol/L)	1.06 ± 0.49	1.14 ± 0.59	1.65 ± 1.48^ad^	1.65 ± 1.15^ad^	0.000
Fasting glucose (mmol/L)	5.55 ± 0.99	5.40 ± 0.96	5.78 ± 1.43	6.07 ± 2.47^d^	0.141
Creatinine (umol/L)	99.68 ± 29.38	118.68 ± 45.27^a^	94.88 ± 21.36^c^	103.15 ± 26.48^d^	0.002
Urinary nitrogen (mmol/L)	6.14 ± 1.37	6.98 ± 2.98^b^	5.69 ± 2.16^c^	6.31 ± 2.09	0.026

Data are presented as mean ± SD unless otherwise indicated. BMI: body mass index; SBP: systolic blood pressure; DBP: diastolic blood pressure; CEA: carcinoembryonic antigen; CA19-9: carbohydrate antigen 19-9.

^a^Significantly different from normal control group (*P* < 0.01). ^b^Significantly different from normal control group (*P* < 0.05). ^c^Significantly different from right colorectal polyps group (*P* < 0.01). ^d^Significantly different from right colorectal polyps group (*P* < 0.05). ^e^Significantly different from left colorectal polyps group (*P* < 0.01).

**Table 2:** The results of binary logistic regression analysis to colorectal polyps.

	*β*	SE	Wald	*P* value	OR	95.0% CI	
						Lower	Upper
Age (years)	−0.034	0.049	0.498	0.480	0.966	0.878	1.063
Female (*n*)	−0.671	1.322	0.257	0.612	0.511	0.038	6.82
BMI (kg/m^2^)	0.495	0.242	4.197	0.040	1.641	1.022	2.635
Hypertension (*n*)	−0.006	0.080	0.006	0.939	0.994	0.849	1.163
SBP (mmHg)	−0.162	0.058	7.722	0.005	0.850	0.758	0.953
DBP (mmHg)	0.046	0.069	0.457	0.499	1.048	0.916	1.199
CEA (ng/mL)	−0.486	0.336	2.092	0.148	0.615	0.319	1.188
CA19-9 (U/mL)	0.011	0.043	0.063	0.801	1.011	0.930	1.099
Total cholesterol (mmol/L)	0.607	0.420	2.088	0.148	1.835	0.805	4.183
Triglycerides (mmol/L)	1.626	1.237	1.727	0.189	5.082	0.450	57.414
Fasting glucose (mmol/L)	0.135	0.683	0.039	0.843	1.144	0.300	4.368
Creatinine (umol/L)	0.005	0.015	0.119	0.730	1.005	0.976	1.035
Urinary nitrogen (mmol/L)	0.470	0.314	2.242	0.134	1.600	0.865	2.962

Abbreviations: CI: confidence interval.
